# Viruses of Respiratory Tract: an Observational Retrospective Study on Hospitalized Patients in Rome, Italy

**DOI:** 10.3390/microorganisms8040501

**Published:** 2020-04-01

**Authors:** Marco Ciotti, Massimo Maurici, Viviana Santoro, Luigi Coppola, Loredana Sarmati, Gerardo De Carolis, Patrizia De Filippis, Francesca Pica

**Affiliations:** 1 Unit of Virology Fondazione Policlinico Tor Vergata, 00133 Rome, Italy; marco.ciotti@ptvonline.it; 2 Department of Biomedicine and Prevention, University of Rome Tor Vergata, 00133 Rome, Italy; maurici@med.uniroma2.it (M.M.); vivianasant@gmail.com (V.S.); patrizia.de.filippis@uniroma2.it (P.D.F.); 3 Clinical Infectious Diseases, Fondazione Policlinico Tor Vergata, 00133 Rome, Italy; luigi.coppolamed@gmail.com (L.C.); sarmati@med.uniroma2.it (L.S.); 4 Health Management, Fondazione Policlinico Tor Vergata, 00133 Rome, Italy; gerardo.decarolis@ptvonline.it; 5 Department of Experimental Medicine, University of Rome Tor Vergata, 00133 Rome, Italy

**Keywords:** influenza, influenza like illness, respiratory viruses, PCR, hospitalized patients

## Abstract

Respiratory tract infections account for high morbidity and mortality around the world. Fragile patients are at high risk of developing complications such as pneumonia and may die from it. Limited information is available on the extent of the circulation of respiratory viruses in the hospital setting. Most knowledge relates to influenza viruses (FLU) but several other viruses produce flu-like illness. The study was conducted at the University Hospital Policlinico Tor Vergata, Rome, Italy. Clinical and laboratory data from hospitalized patients with respiratory tract infections during the period October 2016–March 2019 were analysed. The retrospective analysis included 17 viral agents detected by FilmArray test and clinical data from medical records and hospital discharge sheets. Models were adjusted for relevant confounders such as clinical severity and risk of death, socio-demographic characteristics and surgical procedures. From a total of 539 specimens analysed, 180 (33.39%) were positive for one or more respiratory viruses. Among them, 83 (46.1 %) were positive for influenza viruses (FLU), 36 (20%) rhino/enteroviruses (RHV/EV), 17 (9.44%) human coronaviruses (HCOV-229E, -HKU1, -NL63, and -OC43), 17 (9.44%) respiratory syncytial virus, 15 (8.33%) human metapneumovirus (HMPV), 8 (4.44%) parainfluenza viruses (PIV) and 4 (2.22%) adenoviruses (ADV). The distribution of viral agents varied across age groups and month of detection. The positive specimens were from 168 patients [102 M, 66 F; median age (range): 64 years (19−93)]. Overall, 40% of them had a high-grade clinical severity and a 27% risk of death; 27 patients died and 22 of them (81.5%) had received a clinical diagnosis of pneumonia. Respiratory viral infections may have a severe course and a poor prognosis in hospitalized patients, due to underlying comorbidities. Monitoring the circulation of respiratory viruses in hospital settings is important to improve diagnosis, prevention and treatment.

## 1. Introduction

Infections of the respiratory tract are an important cause of morbidity and mortality around the world. In 2016, about 2.38 million people died because of lower respiratory infections. The highest number of deaths occurred among children younger than 5 years and the elderly. Mortality is particularly high in developing countries [1]. Among the respiratory pathogens, *Haemophilus influenzae* type b, *Streptococcus pneumoniae*, influenza virus and respiratory syncytial virus are the leading causes of lower tract respiratory infections [1]. Although bacterial etiology remains the most common cause of pneumonia, respiratory viral infections are responsible for a large number of hospital admissions among healthy adults, immunocompromised patients, and patients with comorbidities [2,3]. While global monitoring of influenza viruses is well established and aims at the development of effective seasonal vaccines against the influenza virus, the monitoring of other respiratory viruses is not always well defined and requires implementation [4]. From this point of view, the use of rapid, sensitive, multiplex PCR-based tests which allow a broad-spectrum screening of respiratory viruses has clinical relevance. A rapid diagnosis will avoid the administration of an unnecessary antibiotic therapy as well as a prompt isolation of the infected patient, avoiding the spread of the infectious agent between critically ill patients in intensive care units or onco-hematological wards.

Several multiplex PCR-based assays have been approved for in vitro diagnostics of respiratory viruses: BioFire FilmArray Respiratory Panel (bioMerieux Italia, Bagno a Ripoli, Florence, Italy), the GenMark eSensor Respiratory Viral Panel (Carlsbad, CA, USA), xTAG Respiratory Viral panel (Austin, Texas, USA), Allplex™ Respiratory Panel 1–3 (Seegene, Seoul, South Korea), etc. [2,5,6,7].

Here, we present data on the epidemiology of a wide spectrum of respiratory viruses identified in the respiratory specimens of adult hospitalized patients with respiratory symptoms, using the BioFire FilmArray Respiratory Panel v. 1.7. The outcome of viral respiratory infections has been evaluated in relation to the underlying clinical conditions.

## 2. Material and Methods

### 2.1. Clinical Specimens and Study Description

This observational retrospective study was conducted at the University Hospital Policlinico Tor Vergata (PTV) in Rome, Italy. The aim of our study was to evaluate the circulation of respiratory viruses in the period October 2016–March 2019 and in the specific hospital context not only for epidemiological purposes but also in relation to the demographic and clinical characteristics of the infected individuals as well as to their clinical outcome. Therefore, the study included all the hospitalizations of patients with a suspected clinical diagnosis and a documented laboratory diagnosis of respiratory viruses’ infection in the indicated period of time.

Laboratory testing was carried out on clinical request during an ordinary hospitalization—code 1 variable “hospitalization regimen”—in different operative units (OU) of the PTV with a discharge included in the period of time under investigation. Patients under 18 years of age and names associated with non-ordinary hospitalization (code 2—day hospital, code 3—home treatment, code 4—day surgery with overnight stay) were excluded from the analysis.

Clinical and laboratory data of the patients whose respiratory specimens were positive for the presence of one or more respiratory viruses were obtained from the respective medical records based on the availability of data in digitalized clinical archives. A list of predefined variables, such as demographic data, admission and discharge departments, underlying diseases or comorbidities, reasons for admission and surgical interventions, were analysed. The extraction of clinical data was carried out also by analysing hospital discharge sheets through the database of the Hospital Informative System. These data were prepared using the AREAS-ADT information system, using the classification of the ICD-9-CM codes (2007 version) (http://www.salute.gov.it/imgs/C_17_pubblicazioni_2251_allegato.pdf). The resulting layout was subsequently processed for the evaluation of the quantitative and qualitative variables and of the 3M APR-DRG^®^ (All Patient Refined-Diagnosis Related Groups) v. 30.0, that assign to each case a subclass severity of illness or of risk of mortality. The database also included the following variables, that were measured less than 48 h after the respiratory sample collection: flu-like symptoms or other relevant symptoms, presence of fever, hemoglobin values, leukocyte formula and C Reactive Protein (CRP) values.

### 2.2. Laboratory Testing

The respiratory specimens examined in the study included nasopharyngeal swabs, bronchial aspirates (BAS) and bronchoalveolar lavage (BAL) fluids. Respiratory viruses were detected by using the FilmArray Respiratory Panel v. 1.7 (Biomerieux Italia, Bagno a Ripoli, Italy). The assay detects 17 viruses (adenovirus, coronavirus 229E, coronavirus HKU1, coronavirus NL63, coronavirus OC43, human metapneumovirus, influenza A, influenza A subtype H1, influenza A subtype H3, influenza A subtype H1-2009, influenza B, parainfluenza virus 1, parainfluenza virus 2, parainfluenza virus 3, parainfluenza virus 4, human rhinovirus/enterovirus, respiratory syncytial virus) and 3 bacteria (*Bordetella pertussis*, *Chlamydophila pneumoniae*, and *Mycoplasma pneumoniae*). The FilmArray Respiratory Panel consists of an automated extraction of nucleic acids, reverse-transcription, nucleic acid amplification, and melting curve analysis of the PCR product followed by the automatic generation of the report. In a valid run, for each target, the result is reported as “detected” or “not detected”. In case the internal control fails, the result is reported as “invalid” for all analytes included in the panel. The report is obtained in less than 1 h.

### 2.3. Ethics Statement

The study was performed according to the Declaration of Helsinki and in accordance with the International Conference on Harmonization Good Clinical Practice Guidelines [ICH-GCP E6 (R2)]. The Independent Ethical Committee of the University of Rome Tor Vergata approved the study protocol (Approval No. 216/19, December 18th, 2019). All the patients had provided written informed consent before participating in any study-related activities. In respect of privacy, the data were made anonymous by inserting them into databases in Excel format and equipped with an encrypted key available only to the investigators involved in the study. A database was created which contained the nosological number and all the variables under study, with the exception of name and surname. The data were then exported in SPSS v.22.0 format (SPSS Inc., Chicago, Illinois, USA) for statistical analysis.

### 2.4. Statistical Analyses

First of all, we created a unique database (DB) merging two DBs: the laboratory and the hospital ones (the latter one derived from Hospital discharge sheets). Two of us also consulted medical records, when available, in order to gather relevant clinical information (including symptoms) and to enrich information in our database. Consequently, we analysed specific viral agents by month of diagnosis, grouping months in 2 subgroups, cold and not cold months (from November to February and from March to October, respectively). Then we grouped the same class of viruses by the FilmArray assay. Demographic and clinic characteristics, as gender, age groups (18–45, 46–64, ≥65), type of specimens and ward of admission, mode of discharge, including severity of illness and risk of death (according to 3M APR-DRG v.30.0) from I-less to IV-more serious were calculated for all the positive patients. We also analysed single viral agent both for each age group of the patients and for specific symptoms according what found in the clinical documentation. Odds ratios (OR) and 95% confidence intervals (95% CI) were calculated in order to evaluate the association between relevant variable (i.e., females vs males, age, period of diagnosis, severity of illness (SOI), risk of death (ROD), pneumonia, bacterial pneumonia, death, hemoglobin, civil status, education, length of stay (LOS), surgical procedure (according to surgical APR-DRG), major diagnostic category (MDC)) to the two most represented viral infections at the univariate level. The variables that were found to be associated at the univariate level with a *p*-value ≤ 0.15 were included in a multivariate model to adjust the association with the specific viral infection (i.e., the model was used both for influenza and rhino/enterovirus, since they were the two most represented viral infections in our positive patients).

## 3. Results

### 3.1. Circulating Viruses

In this retrospective study, a total of 539 respiratory specimens collected at the University Hospital Policlinico Tor Vergata in the period October 2016–March 2019 were examined. Among them, 180 (33.39% of the total) were positive for the presence of one or more respiratory viruses, whereas in the remaining 359 specimens (66.6% of the total) no virus was detected or the virus was not identified. The number of positive samples by specific viral agent and month of diagnosis is shown in Table 1.

Influenza viruses were the most prevalent organisms detected (83 positive samples, 46.1% of the total), with a marked predominance of the type A (Flu A) with respect to the type B (Flu B) (65 vs. 18 positive samples, respectively, equal to 78.3% vs. 21.7% of the FLU positive samples) (Table 1). Flu B virus was detected only in 2017 and 2018, with a large majority of positive specimens (16 out of 18 total positive samples) in 2018. As expected, the peak of positivity for influenza viruses was observed in the winter, with a maximum of cases (84.33%) in January and February (Table 1). The second most prevalent organisms identified during this study, were rhino/enteroviruses (RHV/EV, 36 isolates, 20% of the positive samples), that were detected in almost all months of the examined years.

Taken together, human coronaviruses (HCOV-229E, -HKU1, -NL63, and -OC43) were the third most prevalent pathogens detected (17 isolates, 9.44% of the positive samples), mainly from December to March (Table 1). The same place was shared with respiratory syncytial virus (RSV) (17 isolated) which was mostly detected in January and February. Considering the remaining respiratory viruses, the observed detection rates were as follows: 15 positive samples (8.33% of the total) for human metapneumovirus (HMPV), 8 (4.44% of the total) for parainfluenza virus (PIV) and 4 (2.22% of the total) for adenovirus (ADV). The details of the different viral agents detected in the examined clinical specimens are shown in Table 2.

### 3.2. Study Population

The 180 samples tested positive for the presence of respiratory viruses were from 168 patients admitted to different wards of the University Policlinico Tor Vergata, in the period of time under examination. Some of them indeed underwent repeated sample collection during hospitalization. Demographics of the 168 positive patients, the type of clinical specimens analysed and their hospital wards of origin are reported in Table 3.

Males and females represented 60.7% and 39.3% of the positive patients, respectively. Their median age was 64 years (range: 19–93 years). Most of them were from the hospital wards of Medicine (43.5%) and Infectious Diseases (40.5%). About three quarters of their clinical specimens consisted of nasopharyngeal swabs, while the remaining specimens included bronchial aspirates (BAS) and broncho-alveolar lavage (BAL) fluids. 

The number of positive samples, by specific viral agent and age class of the patients, is shown in Table 4. It can be observed that the proportion of FLU positive samples was higher in individuals over 64 years old than in the younger ones, whereas the opposite trend was observed for rhino/enteroviruses, whose prevalence was higher in individuals under 65-years old.

### 3.3. Clinical Characteristics and Outcome

Clinical and laboratory data of the patients who were positive for the presence of one or more respiratory viruses in their secretions were obtained from the respective medical records, based on their availability in digitalized clinical archives. In the first analysis, 112 out of 168 medical records, which accounted for 67% of the study population, were examined. Among these 112 individuals, 50 (44.6%) were immunosuppressed, whereas the remaining 62 (55.4%) showed normal immunological parameters. The evaluation of the immune status was performed on the basis of the available clinical and laboratory data, such as clinical diagnosis, hospital ward of admission, blood cells count and pharmacological treatments (i.e., chemotherapy, high-dose steroids and other immunosuppressant; data not shown). Most of influenza viruses were detected in immunocompetent patients, whereas rhino/enteroviruses were mainly found in the immunocompromised (Table 5). The percentages reported in Table 5 refer to the number of patients per group. It is worth noting that there were five coinfections in four immunocompetent patients (three individuals with two viruses and one with three viruses) and four coinfections in four immunocompromised patients (four individuals with two virus each).

Table 6 shows the type and frequency of signs and symptoms from the medical records of 112 positive patients, in relation to the specific viral agents. It can be seen that a total of 121 viruses were detected in 112 patients because some of them tested positive for two or three respiratory viruses (co-infections). Although the numbers do not allow for more in depth analyses, it can be seen that, as expected, fever, dyspnea and cough were the prevalent signs and symptoms reported by the patients. Fever was common in most viral infections, especially in HCOV, PIV and HMPV, slightly less in FLU, RHV/EV and Adeno. In RSV, only 50% had fever, but 83.3% presented with dyspnea, a symptom that was found at high percentages also in other viral infections, i.e., HMPV, RHN/EV, FLU and HCOV, less frequently in ADV and PIV (Table 6).

The examination of the hospital discharge sheets through the database of the Hospital Informative System allowed us to obtain additional clinical info for a total of 151 positive patients, who represented about 90% of our study population. It was thus seen that a large number of patients (60.3%) who tested positive for one or more respiratory viruses (91 out of 151) had received a clinical diagnosis of pneumonia that was based on the presence of fever, symptoms and signs of pneumonia syndrome, the presence of pulmonary infiltrates on chest X-rays or CT scans and virus detection in respiratory specimens. In more detail, 100 clinical diagnoses of pneumonia (91 as principal diagnosis and nine as secondary diagnosis) were reported in 91 positive patients. There were 23 viral pneumonia (25.3% of the total; 11 FLU, 2 HCOV, 1 CMV, 1 HMPV, 1 RSV, 1 PIV, 1 RHV/EV and 5 not-classified), 22 bacterial pneumonia (24.2% of the total; six *Streptococcus pneumoniae*, one *Haemophylus influenzae*, two *Pseudomonas aeruginosa*, four *Staphylococcus aureus*, two *Mycobacterium tuberculosis*, seven non-specified), 12 fungal pneumonia (13.2% of the total; 10 *Aspergillus*, 2 *Pneumocystis jiroveci*) and 43 non-specified pneumonia (47.3% of the total). Bacterial and fungal co-detection were observed particularly in FLU and RHV/EV infections, respectively (data not shown).

The outcome of viral respiratory infections was also evaluated in relation to the underlying clinical conditions of the patients that tested positive for respiratory viruses. Ninety-one patients (60.3 % of the total) presented with low grade severity, the risk of death was found to be elevated only in 41 patients (27.2% of the total) and 27 patients died (18 % of the total patients), as seen in Table 7.

It is worth mentioning that 22 out of the 27 patients who died (81.5%) had received a diagnosis of pneumonia, namely: seven (31.8%) bacterial pneumonia, three (13.6%) viral pneumonia, two fungal pneumonia (9.1%) and 10 (45.5%) not-specified pneumonia.

The crude and adjusted OR (and 95% CI) for variables potentially associated with positivity for FLU and RHV/EV are reported in Table 8 and Table 9, respectively.

Univariate analysis showed that FLU positivity was more likely to be associated with increased age (≥65 years old, *p* = 0.004), months of diagnosis (cold month—*p* < 0.0001), pneumonia for all causes (*p* = 0.008) and Surgical APR-DRG (*p* = 0.032), while length of stay in hospital was not significant either in univariate and multivariate analysis (>19 days—*p* = 0.095 and *p* = 0.117, respectively). Multivariate analysis confirmed a statistically significant existence of a positive correlation only with older age (O.R. 2.9—*p* = 0.028) and diagnosis in the winter months (O.R. 16.08—*p* < 0.0001) (Table 8). Considering RHV/EV, both univariate and multivariate analysis indicated a unique statistically significant correlation with the months of diagnosis, meaning a high probability of detecting RHV/HEV outside of the cold period [O.R. 5.55 (i.e., the reciprocal value of O.R. 0.18); *p* < 0.0001] (Table 9).

## 4. Discussion

Herein, data from a retrospective observational study on the circulation of respiratory viruses in the University Hospital Tor Vergata in Rome, from October 2016 to March 2019, are reported. Epidemiology, demographic characteristics and the clinical outcome of patients tested positive for the presence of one or more respiratory viruses are discussed.

Considering the total number of clinical specimens sent to the Virology Lab for respiratory virus detection during the observation period, the percentage of those tested positive was 33.39%, in agreement with the positivity range reported in similar national and international studies [1,2,3,4].

The identified viral agents included all the respiratory viruses commonly circulating in the community as well as in the hospital setting, namely FLU A and B viruses, RHV/EV, HCOV, RSV, HMPV, PIV and ADV, as already reported by other authors [8,9,10]. It is well known that the knowledge and monitoring of the prevalence of respiratory viruses in hospital settings is very important, as the impact of these infections in hospitalized patients can have more serious consequences than in the general population [1,2,3,4,8,9,10,11,12,13,14,15,16,17].

In our study, 46.1% of the samples tested positive for the presence of influenza A and B viruses, with a greater prevalence of the former over the latter, as also reported in other European countries [18]. As expected, the highest peak of FLU A and B circulation was observed in the cold months (O.R. 16.08—*p* < 0.0001) and in the subjects over 65 years old (O.R. 2.9—*p* = 0.028). The high positivity of the elderly for influenza viruses indicates that an adequate level of vaccination coverage has not yet been reached in this category of individuals. Therefore, they are exposed to the risk of developing severe complications, which are often associated with a poor outcome and require hospitalization, with a consequent increase in public healthcare expenditure. Recent evidence that vaccination coverage is also under the optimal level among hospital physicians and nurses has been recently reported, indicating an additional problem in hospital settings [19].

RHV/EV were, by number, the second group of viruses identified in our respiratory specimens. They showed a wide distribution over the various months of the year with a greater probability of positivity observed in the non-cold months (O.R. 5.55—*p* < 0.0001). They were more frequently found in immunocompromised than in the immunocompetent patients, as reported also in the literature [20,21]. It is known that rhinoviruses (RHV) and respiratory enteroviruses (EV) are the leading causes of upper respiratory tract infections and are among the most frequent infectious agents in humans worldwide. Both are classified in the Enterovirus genus within the *Picornaviridae* family and they have been assigned to seven distinct species, RV-A, B, C and EV-A, B, C, D [21]. In addition to an established etiological role in localized self-limiting diseases, such as the common cold, these viruses demonstrate an unexpected capacity to spread to other body sites under certain conditions and have been identified also as the etiologic agent of severe pneumonia in the elderly and immunocompromised patients, other than of exacerbations of chronic obstructive pulmonary disease and asthma [20,21].

RSV and HCOV were the third most prevalent viruses detected in our hospitalized patients. In our study, RSV and FLU circulated in the same periods, owing to epidemiological interference, although the peak of RSV circulation is described as occurring earlier in other reports [14]. The general distribution of RSV positivity was similar in the different age groups (children were not present in our study population) but dyspnea was documented in 70% of people over 64 years old, in 20% of those in the range 45–64 years old and only in 10% of the younger patients (data not shown), confirming a more severe clinical impact of this infection in the elderly. It is worth mentioning that marked dyspnea was found in over-64-year-old patients also in case of positivity for other respiratory viruses, i.e., MPV (50%), RHN/EV (55%), FLU (69%) and HCOV (50%). In agreement with the potential severity of RSV infection, a recent prospective observational study conducted in France during three influenza seasons revealed that 4% of adults hospitalized for influenza-like illness (ILI) had RSV infection, of whom 58% developed cardiopulmonary complications and 8% died. It also showed that elderly individuals and patients with cancer and/or treated with immunosuppressive drugs are more likely to have RSV isolated when hospitalized for ILI [14].

As mentioned above, human coronaviruses (HCOV), that were represented in our clinical specimens by the strains HCOV-OC43, HCOV-229E, HCOV-HKU1 and HCOV-NL63, showed a positivity rate similar to those of RSV. HCOV can cause enteric or respiratory disease, which can be severe and life threatening in humans, as observed in the case of the zoonotic coronaviruses causing severe acute respiratory syndrome (SARS) and Middle East Respiratory Syndrome (MERS) or in the recent global pandemic caused by the novel coronavirus classified as SARS-CoV-2 and responsible for the respiratory disease named COVID-19 (Corona Virus Disease) [22,23]. Coronaviruses have been associated also with demyelinating diseases [24,25].

It has been proposed recently that coronavirus–host interactions are key to viral pathogenesis and will ultimately determine the outcome of infection. Therefore, there is an increasing interest in unravelling the mechanisms of coronavirus-host interactions at the level of the infected cell, with special attention for the assembly and function of the viral RNA-synthesizing machinery and the evasion of cellular innate immune responses [26].

In our study population, HMPV, known to be a leading cause of lower respiratory tract infection not only in pediatric but also in geriatric populations [11], circulated all year around and showed a distribution in all the age groups, although a higher prevalence in patients over 64 years old was observed. In addition, as reported above, our HMPV positive patients over 64 years old presented with more dyspnea than the younger ones (50% versus 42%).

The positivity rate of ADV and PIV in our clinical specimens was lower than those of the other respiratory viruses. Adenoviruses are DNA viruses that typically cause mild infections involving the upper or lower respiratory tract, the gastrointestinal (GI) tract, or conjunctiva; however, it has been reported that ADV-associated diseases are more severe and dissemination is more likely in patients with impaired immunity [27,28]. Similarly, the paramyxovirus family may evoke lower respiratory infections in infants, children and immunocompromised individuals. Among non-immune-compromised adults, HPIV infection typically causes mild disease characterized by upper respiratory tract symptoms and it is infrequently associated with severe croup or pneumonia. HPIV infection may be associated also with viral exacerbations of chronic airway diseases, asthma or COPD or chronic rhinosinusitis [29].

It is of note that, overall, 40% of our study population presented with a high-grade clinical severity, and a 27% risk of death, as expected in critical ill patients who are exposed to several viral infections and/or reactivation [30,31,32]. Indeed, 27 patients died and 22 of them (81.5%) had received a clinical diagnosis of pneumonia. This is one of the most relevant clinical findings of our study, which underlines the role of pneumonia in the unfavourable outcome of patients tested positive for respiratory viruses. On the epidemiological side, the main result of Our The main finding our multivariate model confirms that FLU viruses circulate mainly in the elderly and during cold months, unlike RHV/EV, that show a higher probability to be detected in non-cold months, irrespective of the age of the patients.

This study has some limitations, in part related to the difficulty to obtain from digital archives complete clinical information for all the patients tested positive for respiratory viruses. This limitation is due to the retrospective nature of the study and is common to all other similar studies. Another limitation is represented by the time interval under examination (i.e., October 2016–March 2019) which, in consideration of the seasonal circulation of some respiratory viruses, did not allow us to collect complete data on the circulation of some of them (e.g., ADV and HMPV not detected in 2019, PIV and RSV in 2016, RHV/EV in 2019).

Despite these limitations, Espandi our findings contribute to underlining the relevant impact of the respiratory viruses positivity on the clinical outcome of hospitalized patients, also in relation to their underlying clinical conditions. Viral infections of the respiratory tract pose a high burden in both hospitalized patients and in the community. Several risk factors exist for severe viral respiratory disease, but many severe respiratory tract infections cannot be explained only by these risk factors. Recently, a growing body of evidence shows that the composition of the airway’s microbiota has a major influence on the training of both mucosal and systemic immune responses and thus may influence susceptibility to severe viral infections [33]. Therefore, the influence of bacterial colonization or genetic factors on the severity of viral respiratory infections needs further in-depth investigations [34,35].

Another crucial point relates to the fact that studies in humans and animal models have shown that in respiratory viral infections, symptoms are not immediate and appear days or even weeks after infection [35]. Considering that the initial symptoms represent the manifestation of virus recognition by the host innate immune system, it can be speculated that viruses are left free to multiply undisturbed for a long period of time. This fact is clinically and epidemiologically relevant as it highlights the ability of some respiratory viruses to elude the innate antiviral immune response in the early stages of infection, with consequent high viral multiplication in the infected people and increased possibility of infecting contacts [35].

In conclusion, based on the above findings, the adequate epidemiological surveillance of respiratory viruses infections is highly recommended and mandatory in hospital settings, to improve the diagnosis, therapy and prognosis of hospitalized patients. In addition, flu vaccination of all people at risk and care staff must be pursued with a greater determination, in light of recent epidemiological evidence [36,37,38,39]. We feel that we must prepare to face the consequences of new possible epidemics from other respiratory viruses and we must learn to know them better and to discriminate between the different virus types and their clinical consequences [40]. Finally, there is a need for experimental models to get deeper insights about the complex virus–host interactions that enable us to undertake the best prevention and control strategies in hospital settings and in the community.

## Figures and Tables

**Table 1:** Number of positive samples by specific viral agent and month of diagnosis in the whole period of observation.

Month	ADV No. (%)	PIV No. (%)	MPV No. (%)	RSV No. (%)	HCOV No. (%)	HRV/EV No. (%)	FLU A No. (%)	FLU B No. (%)	Total No. (%)
January	−	2 (3.6)	4 (7.1)	11 (19.6)	3 (5.4)	3 (5.4)	21 (37.5)	12 (21.4)	56 (31.1)
February	1 (2.0)	1 (2.0)	2 (4.1)	1 (2.0)	3 (6.1)	4 (8.2)	34 (69.4)	3 (6.1)	49 (27.2)
March	−	−	2 (12.5)	2 (12.5)	5 (31.3)	3 (18.8)	3 (18.8)	1 (6.3)	16 (8.9)
April	1 (12.5)	1 (12.5)	1 (12.5)	−	2 (25.0)	3 (37.5)	−	−	8 (4.4)
May	1 (16.5)	−	2 (33.3)	1 (16.7)	−	2 (33.3)	−	−	6 (3.3)
June	−	−	−	−	−	2 (100.0)	−	−	2 (1.1)
July	−	2 (50.0)	1 (25.0)	−	−	1 (25.0)	−	−	4 (2.2)
August	−	−	−	−	−	−	−	−	0 (0.0)
September	−	−	−	−	−	−	−		0 (0.0)
October	−	−	−	−	1 (25.0)	3 (75.0)	−	−	4 (2.2)
November	1 (10.0)	−	1 (10.0)	−	−	8 (80)	−	−	10 (5.6)
December	−	2 (8.0)	2 (8.0)	2 (8.0)	3 (12.0)	7 (28.0)	7 (28.0)	2 (8.0)	25 (13.9)
Total	4 (2.2)	8 (4.4)	15 (8.3)	17 (9.4)	17 (9.4)	36 (20)	65 (36.1)	18 (10.0)	180 (100)

**Table 2:** Detection of respiratory viruses by FilmArray assay.

Virus	No. (% of the Total Number)	Total Number per Virus Type No. (% of the Total Number of Positives)
Coronavirus-HKU1	3 (17.6)	17 (9.4)
Coronavirus-OC43	6 (35.3)	
Coronavirus-229E	3 (17.6)	
Coronavirus-NL63	5 (29.4)	
Influenza Virus A H1–2009	27 (41.5)	65 (36.1)
Influenza Virus A H3	35 (53.8)	
Influenza Virus A	3 (4.6)	
Influenza Virus B	18 (100.0)	18 (10.0)
Rhino/Enterovirus	36 (100.0)	36 (20.0)
Parainfluenza Virus 3	6 (75.0)	8 (4,4)
Parainfluenza Virus 2	1 (12.5)	
Parainfluenza Virus 4	1 (12.5)	
Metapneumovirus	15 (100.0)	15 (8.3)
Adenovirus	4 (100.0)	4 (2.2)
Respiratory Syncytial Virus	17 (100.0)	17 (9.4)

**Table 3:** Demographic and clinical characteristics of the positive patients.

		No. (%)
Gender	Male	102 (60.7)
	Female	66 (39.3)
Age Groups (years)	18–45	25 (14.9)
	46–64	59 (35.1)
	≥65	84 (50.0)
Type of samples	Nasopharyngeal swab	125 (74.4)
	BAS/BAL	43 (25.6)
Wards of admission	Haematology	6 (3.6)
	Infectious Diseases	68 (40.5)
	Trasplant Unit	14 (8.3)
	Medicine	73 (43.5)
	Surgery	7 (4.2)
Total		168 (100.0)

BAS/BAL: bronchial aspirate/broncho-alveolar lavage.

**Table 4:** Number of positive samples by specific viral agent and age class of the patients.

Age (years)	No.	ADV No. (%)	PIV No. (%)	HMPV No. (%)	RSV No. (%)	HCOV No. (%)	RHN/EV No. (%)	FLU A No. (%)	FLU B No. (%)
18–45	25	2 (8.0)	3 (12.0)	2 (8.0)	3 (12.0)	1 (4.0)	6 (24.0)	11 (44.0)	1 (4.0)
46–64	59	2 (3.4)	2 (3.4)	5 (8.5)	5 (8.5)	8 (13.6)	16 (27.1)	16 (27.1)	4 (6.8)
≥ 65	84	0 (0.0)	3 (3.6)	8 (9.5)	9 (10.7)	8 (9.5)	14 (16.7)	38 (45.2)	13 (15.5)
Total	168	4 (2.4)	8 (4.8)	15 (8.9)	17 (10.1)	17 (10.1)	36 (21.4)	65 (38.7)	18 (10.7)

**Table 5:** Positivity per specific viral agent and immune-competence status.

Virus	Immunocompetent Patients No. (% of Patients)	Immunocompromised No. (% of Patients)
Adenovirus	2 (3.2)	2 (4.0)
Parainfluenza Virus	2 (3.2)	5 (10.0)
Metapneumovirus	8 (12.9)	7 (14.0)
Respiratory Syncytial Virus	8 (12.9)	4 (8.0)
Coronavirus	2 (3.2)	8 (16.0)
Rhino/Enterovirus	9 (14.5)	19 (38.0)
Influenza Virus	36 (58.1)	9 (18.0)
Viral Coinfections	*5 (6.5)*	*4 (8.0)*
Total Patients	62 (100)	50 (100)

**Table 6:** Number of samples by specific viral agent and sign and symptom.

Sign/Symptom	No. (%)						
	ADV 4 (100)	PIV 7 (100)	HMPV 15 (100)	RSV 12 (100)	HCOV 10 (100)	RHN/EV 28 (100)	FLU 45 (100)
Fever	3 (75)	7 (100)	13 (86.7)	6 (50)	8 (80)	17 (60.7)	34 (75.6)
Dyspnea	2 (50)	4 (57.1)	12 (80)	10 (83.3)	6 (60)	20 (71.4)	29 (64.4)
Dry cough	0 (0)	3 (42.9)	2 (13.3)	1 (8.3)	2 (20)	9 (32.1)	9 (20)
Productive cough	1 (25)	2 (28.6)	5 (33.3)	3 (25)	1 (10)	5 (17.9)	15 (33.3)
Sore throat	0 (0)	1 (14.3)	2 (13.3)	0 (0)	0 (0)	3 (10.7)	7 (15.6)
Nasal congestion	0 (0)	1 (14.3)	1 (6.7)	1 (8.3)	1 (10)	2 (7.1)	2 (4.4)
Myalgia	1 (25)	1 (14.3)	0 (0)	0 (0)	1 (10)	3 (10.7)	4 (8.9)
Headache	1 (25)	1 (14.3)	2 (13.3)	1 (8.3)	1 (10)	2 (7.1)	2 (4.4)
Schivers	1 (25)	1 (14.3)	3 (20)	0 (0)	1 (10)	2 (7.1)	2 (4.4)
Joint pain	3 (75)	1 (14.3)	0 (0)	0 (0)	2 (20)	4 (14.3)	1 (2.2)
Retrosternal pain	0 (0)	0 (0)	4	2 (16.7)	1 (10)	4 (14.3)	6 (13.3)
Sweating	1 (25)	2 (25)	0 (0)	1 (8.3)	0 (0)	1 (3.6)	4 (8.9)
Abdominal pain	0 (0)	0 (0)	1 (6.7)	0 (0)	2 (20)	1 (3.6)	7 (15.6)
Nausea	1 (25)	2 (28.6)	1 (6.7)	1 (8.3)	1 (10)	2 (7.1)	3 (6.7)
Vomiting	0 (0)	1 (14.3)	1 (6.7)	1 (8.3)	1 (10)	2 (7.1)	4 (8.9)
Diarrhea	0 (0)	0 (0)	2 (13.3)	0 (0)	1(10)	0 (0)	5 (11.1)

**Table 7:** Clinical severity, risk of death (according to APR-DRG classification system) and modality of discharge of positive patients.

	No. (%)
***Clinical Severity*** **(APR-DRG)**	
I (Minor)	24 (15.9)
II (Moderate)	67 (44.4)
III (Major)	46 (30.5)
IV (Extreme)	14 (9.3)
***Risk of death*** **(APR-DRG)**	
I (Minor)	60 (39.7)
II (Moderate)	50 (33.1)
III (Major)	30 (19.9)
IV (Extreme)	11 (7.3)
***Mode of discharge***	
At home	76 (50.3)
Home self-care with planned readmission	33 (21.9)
Death	27 (17.9)
At other care facilities	11 (7.3)
On voluntary basis	4 (2.6)
**TOTAL**	*n* = 151 (100.0%)

**Table 8:** Crude and Adjusted OR (and 95% CI) of Influenza, according to relevant variables.

Variables	O.R.	95% C.I.	*P*	Adjusted for Variables *	95% C.I.	*p*
Females vs Males	0.74	0.34–1.39	0.355			
Age ≥ 65 yearsold	2.46	1.32–4.59	**0.004**	**2.9**	**1.10–5.61**	**0.028**
Period of diagnosis (cold period ^)	21.94	7,37–65,33	**<0.0001**	**16.08**	**5.17–54.61**	**<0.0001**
APR-DRG Severity of illness (SOI) high (3–4)	0.85	0.44–1.62	0.616			
APR-DRG Risk of death (ROD) high (3–4)	0.75	0.36–1.53	0.425			
Pneumonia (all causes)	0.40	0.21–0.79	**0.008**	0.47	0.20–1.12	0.089
Bacterial Pneumonia	0.482	0.19–1.23	**0.120**	0.89	0.23–3.48	0.869
Death	1.45	0.62–3.38	0.383			
Haemoglobin (<10)	0.70	0.37–1.33	0.329			
Civil status (Married)	1.19	0.54–2.60	0.694			
Education (high)	0.49	0.16–1.52	0.277			
^†^ LOS (>19 days)	1.82	0.94–3.55	**0.095**	2.08	0.90–4.88	0.091
Surgical procedure (Surgical APR-DRG)	2.62	1.07–6.42	**0.032**	1.85	0.63–5.47	0.264
MDC (04 = pulmonary)	0.88	0.46–1.66	0.690			

* *p*-value < 0.20 at univariate level; ^ cold period: Dec, Jan, Feb; SOI = severity of illness; ROD = risk of death; † length of stay.

**Table 9:** Crude and Adjusted OR (and 95% CI) of Rhino-Entero virus, according to relevant variables.

Variables	O.R.	95% C.I.	*p*	Adjusted for Variables *	95% C.I.	*P*
Females vs Males	1.34	0.63–2.82	0.443			
Age ≥ 65 years old	**0.57**	**0.27–1.21**	**0.143**	0.65	0.27–1.59	0.352
Period of diagnosis (cold period ^)	**0.165**	**0.07–0.36**	**<0.0001**	**0.18**	**0.07–0.44**	**<0.0001**
APR-DRG (SOI) high (3–4)	1.08	0.49–2.41	0.84			
APR-DRG (ROD) high (3–4)	1.65	0.71–3.84	0.242			
Pneumonia (all causes)	1.50	0.65–3.46	0.340			
Bacterial Pneumonia	**2.04**	**0.75–5.55**	**0.156**	1.64	0.49–5.55	0.423
Death	1.46	0.55–3.84	0.444			
Haemoglobin (<10)	1.35	0.63–2.87	0.439			
Civil status (Married)	0.97	0.37–2.53	0.955			
Education (high)	1.07	0.28–4.08	0.919			
^†^ LOS (>19 days)	**0.49**	**0.20–1.19**	**0.117**	0.45	0.17–1.23	0.119
Surgical procedure	0.62	0.20–1.96	0.417			
MDC (04 = pulmonary)	1.14	0.52–2.51	0.745			

* *p*-value < 0.20 at univariate level; ^ cold period: Nov. Dec, Jan, Feb; SOI = severity of illness; ROD = risk of death; † length of stay.
